# Social Distancing: Prevalence of Depressive, Anxiety, and Stress Symptoms Among Brazilian Students During the COVID-19 Pandemic

**DOI:** 10.3389/fpubh.2020.589966

**Published:** 2021-01-27

**Authors:** Cristiane Silva Esteves, Camila Rosa de Oliveira, Irani Iracema de Lima Argimon

**Affiliations:** ^1^Instituto Federal de Educação, Ciência e Tecnologia do Rio Grande do Sul, Porto Alegre, Brazil; ^2^Faculdade Meridional IMED, Passo Fundo, Brazil; ^3^Pontifícia Universidade Católica do Rio Grande do Sul, Porto Alegre, Brazil

**Keywords:** COVID-19, mental health, students, social distancing, pandemic

## Abstract

Social distancing due to the COVID-19 pandemic can impact mental health, triggering symptoms such as anxiety, stress and depression. Therefore, this study aimed to assess the levels of anxiety, depression and stress during the period of social distancing due to COVID-19 in students from a campus of the Federal Institute in the metropolitan area of Porto Alegre/RS. A correlational and exploratory study was performed. The sample of the present research was composed by 208 students, who responded to a self-administered online questionnaire with sociodemographic variables and the Depression, Anxiety and Stress Scale Short Form – DASS-21. The detected prevalence of symptoms classified as moderate-severe was 49% for stress, 39% for depression and 33% for anxiety. An association was found between higher levels of anxiety symptoms (OR = 5.652; 95% CI = 2.872–11.123; *p* < 0.001), depression (OR = 3.289; 95% CI = 1.810–5.978; *p* < 0.001) and stress (OR = 5.684; 95% CI = 3.120–10.355; *p* < 0.001) with occurrence of sleep problems during the period of social distancing. There was a protective factor provided by regular physical exercise in relation to depressive symptoms (OR = 0.490; 95% CI = 0.250–0.960; *p* =0.033). These data are extremely important for understanding the adverse effect on the mental health of students and for developing psychological support strategies, thus promoting well-being during and after the pandemic.

## Introduction

The new coronavirus (Coronavirus Disease 2019 - COVID-19) caused by the SARS-CoV-2 virus is highly transmissible. The physical symptoms usually manifested are runny nose, cough, sore throat, body aches, nausea, vomiting, diarrhea, fever, and breathing difficulties (1). However, in more severe cases can cause heart damage and respiratory failure and can lead to death (2).

In order to reduce transmission, experts recommend measures of social distancing that have been adopted worldwide, restricting the movement of people (3). Vasconcelos et al. (4) performed a narrative review regarding the effect of the quarantine and reported that social distancing can cause sadness, anger and anxiety.

On the exact day of September 2, 2020, globally 25.541.380 cases were confirmed, including 852.000 deaths reported by the World Health Organization (5). Data from Brazil demonstrate that the country had 3.908.272 confirmed cases of COVID-19 and 121.381 deaths from the disease (5). As a result, Brazil is one of the countries with a high number in the community transmission of the disease (6).

The World Health Organization declared on January 30, 2020, the outbreak caused by COVID-19 constitutes a public health emergency of international importance, and on March 11 it was characterized as a pandemic (5). Faced with a public health emergency such as the COVID-19 pandemic, quarantine and social distancing can impact mental health, triggering symptoms such as anxiety, fear, anger, stress, and depression (4). These mandatory changes in routine to reduce contamination and spread of the virus can impact psychological well-being and mental health (7, 8).

Economic and social repercussions of the pandemic reported worldwide cause insecurity (4, 8). Studies show psychological impacts directly linked to the COVID-19 pandemic, manifested through symptoms of anxiety, stress and depression (9–11).

Schmidt et al. (12) performed a narrative literature review including studies from different countries and reported that studies regarding the impacts on mental health-related to the pandemic are still scarce, as considered a recent phenomenon. For the same reason, Brazilian studies examining the psychological impact of COVID-19 on the Brazilian population are also scarce. Therefore, this study aimed to assess the levels of anxiety, depression, and stress during the period of social distancing due to COVID-19 in students from a campus of the Federal Institute in the metropolitan area of Porto Alegre/RS.

## Materials and Methods

### Design

Correlational and exploratory study.

### Participants

The sample of the present research was composed by 208 students from a campus of the Federal Institute of Education, Science, and Technology of Rio Grande do Sul (*IFRS*) in the metropolitan area of Porto Alegre/RS. The participants were recruited in a non-probabilistic approach and the survey link was sent to the participants via email and shared through messenger apps. Ages ranged from 15 to 64 years (*M* = 26.33; *SD* = 11.94) and 80% were women (*n* = 167). Regarding education, 75% (*n* = 156) were students of technical courses or technical courses integrated to high school, 18% (*n* = 37) of undergraduate courses, and 7% (*n* = 15) of the National Program for the Integration of Professional Education (*PROEJA*). The inclusion criteria included were being enrolled in a course on the *IFRS* campus where the research was conducted. The exclusion criteria were not completing the instruments. The campus has 502 students enrolled and a total of 243 students answered the questionnaire between April 1 and 18, 2020, representing a response rate of 48.4%, however 35 students were excluded due to incomplete answers.

### Instruments

#### Sociodemographic Questionnaire

A **s**elf-administered online questionnaire with sociodemographic variables regarding marital status, age, sex, work activities, physical exercise, study, sleep and social relations and including information on precautionary measures against COVID-19, working habits and conditions during social distancing measures.

#### Depression, Anxiety, and Stress Scale Short Form – DASS-21

DASS-21 was developed by Lovibond and Lovibond (13), a 21-item scale that assesses symptoms of depression, anxiety and stress experienced in the last week. The instrument application results in the classification of symptoms separately and according to their severity. The Brazilian version, entitled *Escala de Depressão, Ansiedade e Estresse (DASS-21)* was adapted and validated by Vignola and Tussi (14) and presents good internal consistency for depression (*a* = 0.92), anxiety (*a* = 0.86) and stress (*a* = 0.90). In 2016, a new Brazilian validation was published by Patias, Lara Machado, Bandeira and Dell'Aglio (15) and the subscales demonstrated adequate levels of internal consistency ranging from 0.83 to 0.90. The cut-off scores for the mild, moderate and severe categories are, respectively, 0–13, 14–20, and 21 or more for depression; 0–9, 10–14, and 15 or more for anxiety; 0–18, 19–25, and 16 or more points for stress.

### Data Collection Procedures

This study was approved by the Research Ethics Committee of the Federal Institute of Education, Science and Technology of Rio Grande do Sul (CAAE: 08694919.1.0000.8024) and authorized by the campus for its performance. Participants were invited electronically to the survey. Data collection occurred online during April 2020, at the beginning of the period of social distancing due to the pandemic of COVID-19. All participants that are over 18 gave their consent. For the participation of underage students, parents and/or guardians signed the Informed Consent Form and the students signed the Informed Assent Form. Parental and guardian consent for underage students was previously obtained in another stage of the research that evaluated the same variables.

### Data Analysis

Descriptive statistics was used as mean, standard deviation and percentage for the presentation of data on the demographic profile of the participants and the scores obtained in the instruments. From the analysis of the Kolmogorov-Smirnov test, which suggested the use of non-parametric techniques, the association between the variables was verified using the Chi-square test. Analyzes were conducted using Statistical Package for the Social Sciences software version 24 for Windows and significant results were considered when *p* < 0.05.

## Results

Regarding the marital status of the participants, 71% (*n* = 147) were single, 24% (*n* = 49) married or in a stable relationship, 4% (*n* = 9) divorced and 1% (*n* = 3) widower. Only one (<1%) of the students had a confirmed diagnosis of COVID-19. Most students performed the social distancing partially (76%, *n* = 159), leaving home to work or for basic needs (such as shopping at the supermarket or pharmacy). Thus, 63% (*n* = 130) of students do not work, 14% (*n* = 32) were on vacation or temporary layoff during the pandemic period, 13% (*n* = 26) had no changes in their work routine and 10% (*n* = 20) started to perform their work activities in the home-office format. According to Table 1, during the period of social distancing, most students reported not practicing physical activities, maintaining engagement in study-related activities, having difficulties sleeping, and maintaining contact with family and friends through the internet or telephone.

**Table 1:** Physical activities, study, sleep and social relationships during social distancing (*n* = 208).

	***n***	**%**
**Weekly practice of physical activity (30 min or more)**		
No	152	73
Yes	56	27
**Maintain study activities**		
No	83	40
Yes	125	60
**Sleep changes**		
No, sleep without difficulties	96	46
Yes, have trouble sleeping	112	54
**Social relationships**		
Do not maintain contact (phone or internet) with family/friends	12	6
Maintain contact (phone or internet) with family/friends	196	94

The mean and standard deviation of the scores obtained in each of the DASS-21 scales are presented in Table 2, as well as the classification of symptom intensity. Considering the moderate and severe intensities, the stress symptoms presented the highest prevalence (49%), followed by depression symptoms (39%) and anxiety symptoms (33%).

**Table 2:** Mean, standard deviation and classification of DASS-21 subscale symptom intensity (*n* = 208).

	***M***	***SD***	**Minimum**	**Maximum**	**Symptom intensity classification**	
					**Minimum/ Mild**	**Moderate/ Severe**
**DASS-21**						
Anxiety	8.23	8.93	0	40	137 (67%)	69 (33%)
Depression	12.03	10.42	0	42	128 (61%)	80 (39%)
Stress	14.24	10.02	0	42	106 (51%)	102 (49%)

According to Table 3, there was an association between higher levels of anxiety symptoms (Chi-Square = 29.337; OR = 5.652; 95% CI = 2.872–11.123; *p* < 0.001), depression (Chi-Square = 16.228; OR = 3.289; 95% CI = 1.810–5.978; *p* < 0.001) and stress (Chi-Square = 35.469; OR = 5.684; 95% CI = 3.120–10.355; *p* < 0.001) with the occurrence of sleep problems during the period of social distancing. There was association between anxiety symptoms and maintenance of study related activities (Chi-Square = 3.935; OR = 1.840; 95% CI = 0.998–3.395; *p* < 0.047). There was an association between weekly practice of physical activity and depressive symptoms (Chi-Square = 4.566; OR = 0.490; 95% CI = 0.250– 0.960; *p* = 0.033), suggesting a protective factor. The OR and CI values indicate that there is no significant relationship between social relationship and anxiety, depression or stress.

**Table 3:** Associations between DASS-21, physical activity, study activities, sleep changes, and social relationships (*n* = 208).

	**DASS-21**														
	**Anxiety**					**Depression**					**Stress**				
	**Min./Mild**	**Moderate/Severe**	**Chi-square**	**OR**	**95% CI**	**Min./Mild**	**Moderate/Severe**	**Chi-square**	**OR**	**95% CI**	**Min./Mild**	**Moderate/Severe**	**Chi-square**	**OR**	**95% CI**
**Weekly practice of physical activity (%)**															
No	97 (70)	55 (80)	2.385	0.588	[0.295, 1.171]	87 (68)	65 (32)	4.566^*^	0.490	[0.250, 0.960]	72 (68)	80 (78)	2.917	0.582	[0.312, 1.087]
Yes	42 (30)	14 (20)				41 (81)	15 (19)				34 (32)	22 (22)			
**Maintain study activities (%)**															
No	62 (45)	21 (30)	3.935^*^	1.840	[0.998, 3.395]	52 (41)	31 (39)	0.072	1.081	[0.611, 1.915]	47 (44)	36 (35)	1.777	1.460	[0.836, 2.553]
Yes	77 (55)	48 (70)				76 (59)	49 (61)				59 (56)	66 (65)			
**Sleep changes (%)**															
No	82 (59)	14 (20)	29.337^*^	5.652	[2.872, 11.123]	73 (57)	23 (29)	16.228^*^	3.289	[1.810, 5.978]	70 (66)	26 (25)	35.469^*^	5.684	[3.120, 10.355]
Yes	57 (41)	55 (80)				55 (43)	57 (71)				36 (34)	76 (75)			
**Social relationships (%)**															
Do not maintain contact	6 (4)	6 (9)	1.537	0.474	[0.147, 1.527]	5 (4)	7 (9)	2.055	0.424	[0.130, 1.385]	5 (5)	7 (7)	0.442	0.672	[0.206, 2.189]
Maintain contact	133 (96)	63 (91)				123 (96)	73 (91)				101 (95)	95 (93)			

*df = 206*.

* *p < 0.05*.

## Discussion

This study aimed to verify the levels of anxiety, depression, and stress during the period of social distancing due to COVID-19 in students of a campus of the Federal Institute of the metropolitan area of Porto Alegre/RS. During disease outbreaks, the anxiety of the population may increase after the first death, and due to the increase of news in the media and the growing number of new cases (16). Other stressors are the fear of being infected, the amount of news, and the uncertainty about the cure of the disease (10). Studies similar to the present study were performed in China, where the COVID-19 epidemic began, which verified the impacts of the COVID-19 outbreak on mental health (9–11).

Wang et al. (11) conducted a survey, similar to this study, with 1.210 Chinese to verify the psychological impact of the COVID-19 pandemic, using the Depression, Anxiety and Stress Scale - Short Form (DASS-21). The data obtained revealed 28.8% of participants with moderate to severe anxiety symptoms, 16.5% with moderate to severe depressive symptoms and 8.1% with intense stress levels. The study also revealed that students had a psychological impact from the COVID-19 outbreak identified by higher levels of stress, anxiety and depression that may have been increased due to uncertainty and the potential negative impact on academic progression (11).

The study by Qian et al. (9) aimed to verify psychological responses to the threat of the Coronavirus among residents of the Chinese cities Shanghai (*n* = 501) and Wuhan (*n* = 510), and found that 20.4 and 32.7% had moderate or severe anxiety, respectively. Thus, the study by Zhang et al. (10) with 1.593 participants identified, through a self-administered online questionnaire, the symptoms of anxiety, depression and stress due to the COVID-19 pandemic were 44.7, 73.4, and 50.7%, respectively.

The different levels of anxiety, depression and stress found in the studies can be explained. The studies were performed at different times of the pandemic, with the participants being involved at the beginning or the peak of the outbreak of COVID-19. The increase in the number of confirmed cases and deaths in the city may also explain the different levels of impact on mental health presented by the studies. In addition, the protective measures adopted by governments may be different, making the population feel more or less protected. In addition, according to Zandifar and Badrfam (17) other factors can lead to psychological distress, such as disruptions in academic activities, career opportunities, financial issues and movement restrictions.

Measures to reduce the contamination and spread of COVID-19 include social isolation of those infected, quarantine of suspected cases or those who have had contact with a confirmed case, and social distancing from other people with reduced contact and face-to-face social interaction. These can be considered potential stressors, which can increase the levels of anxiety, stress and depression.

During periods of confinement at home, the population tends to adopt a sedentary routine, which could lead to psychosocial distress in form of depression and anxiety (18). In our study we found that weekly exercise offers protection against depression. Regular physical exercise is able to improve symptoms of anxiety and depression (19) and amplify positive emotions such as happiness and well-being (20).

Even studies showing different levels of anxiety, depression and stress-related to the outbreak of COVID-19, all the studies mentioned an increase in psychological problems during this pandemic. According to Zandifar and Badrfam (17) may be associated with the severity of the disease and the uncertainty of the cure, as well as not having a clear forecast. Uncertainty brings a feeling of not being able to control the threat from the invisible. Social distancing protects people from imminent contagion, while it can favor certain thoughts and can lead to more anxiety, sadness and stress.

## Final Considerations

The COVID-19 pandemic has brought and will continue to bring economic, social and psychological impacts worldwide (4). The Covid-19 outbreak causes changes in work routines, unemployment, social distancing, closing schools, universities, public places, companies, which interfere in mental and emotional health, causing feelings of helplessness and abandonment (8). Study limitations refer to the predominance of female participants and lack of information about the use of medications.

Therefore, this study presented levels of anxiety, depression, and high stress during the period of social distancing due to the Coronavirus in students from a campus of the Federal Institute of the metropolitan area of Porto Alegre/RS. These data are extremely important for understanding the adverse effect on the mental health of students and for developing psychological support strategies, thus promoting well-being during and after the pandemic.

## Data Availability Statement

The datasets presented in this article are not readily available because Data accessible only to the authors of the study as approved by the ethics committee. Requests to access the datasets should be directed to Cristiane Esteves, crissilvaesteves@gmail.com.

## Ethics Statement

The studies involving human participants were reviewed and approved by Pontifícia Universidade Católica do Rio Grande do Sul. Written informed consent to participate in this study was provided by the participants' legal guardian/next of kin.

## Author Contributions

CE conducted the data collection. CO performed statistical analysis using SPSS. IA advised the research. All authors were responsible for the study design and performed the literature review, and contributed to the article and approved the submitted version.

## Conflict of Interest

The authors declare that the research was conducted in the absence of any commercial or financial relationships that could be construed as a potential conflict of interest.
